# A Comparative Analysis of Microscopic and Endoscopic Stapedotomy: A Prospective Study

**DOI:** 10.1055/s-0045-1811252

**Published:** 2026-03-03

**Authors:** Ishi Jain, Pradeep Singh Rawat

**Affiliations:** 1 Department of Otorhinolaryngology, Subharti Medical College, Swami Vivekanand Subharti University, Meerut, Uttar Pradesh, India

**Keywords:** otosclerosis, dizziness, drilling, stapedotomy, endoscopic

## Abstract

**Introduction:**

Stapedotomy is a procedure performed for the treatment of otosclerosis in which a small hole is created in the footplate stapes and a prosthesis is inserted from the incus to the vestibule.

**Objective:**

The present study aims to compare the benefits of endoscopic stapedotomy with those of microscopic stapedotomy.

**Methods:**

We included 20 patients of the Outpatient Department of Ear, Nose, and Throat (ENT) of a private medical college, who were diagnosed with otosclerosis and were scheduled to undergo stapedotomy. After they provided informed consent, the patients were randomly divided into two groups: group A underwent microscopic stapedotomy, and group B, endoscopic stapedotomy. Both the groups were compared in terms of intraoperative time, surgical approach, visualization of anatomical landmarks, extent of drilling of the posterosuperior part of external auditory canal, manipulation of the facial nerve, and postoperative pain, dizziness, and hearing loss.

**Results:**

A postural approach was required in 4 patients in group A. Visualization of the anatomical landmarks was better in group B. The extent of drilling of the posterosuperior part of the external auditory canal was minimal in group B, whose patients only experienced mild dizziness and low levels of pain postoperatively.

**Conclusion:**

Both techniques demonstrated high efficacy in improving hearing outcomes and reducing postoperative complications. Endoscopic stapedotomy offers enhanced visualization of anatomical landmarks and requires minimal drilling and minimal manipulation of the facial nerve. Moreover, the endoscopic procedure results in lower levels of postoperative pain and dizziness, as well as better cosmetic outcomes.

## Introduction

Stapedotomy is a procedure performed for the treatment of otosclerosis in which a small hole is created in the footplate stapes and a prosthesis is inserted from the incus to the vestibule.

## Objective

The present study aims to compare the benefits of endoscopic stapedotomy with those of microscopic stapedotomy.

## Methods

The current study was conducted at the Outpatient Department of Ear, Nose, and Throat (ENT) of a private medical college between February 2023 and February 2024. After the application of the inclusion and exclusion criteria, we selected 20 cases of otosclerosis to compose the sample. After receiving approval from the institutional Ethics in Research Committee and obtaining proper informed written consent from each patient, the sample was randomly divided into two groups: group A underwent microscopic stapedotomy, and group B, endoscopic stapedotomy. Stapedotomy was performed in all the patients either via the transcanal approach or the postaural approach. A tympanomeatal flap was raised, and the middle ear structures were visualized. Then, the posterior wall of the external auditory canal was drilled, and stapes fixation was checked. After that, the stapedial tendon was dissected, the incudostapedial joint was cut, and the anterior and posterior crura of the stapes were fractured and removed. A small hole, or fenestration, was created in the footplate of the stapes using a microdrill. Then, a teflon prosthesis was inserted into the fenestration to bypass the fixed stapes, the tympanomeatal flap was repositioned, and the external auditory canal was filled with abgel. 5

The inclusion criteria were a diagnosis of otosclerosis based on clinical history, an otoscopic examination within normal limits, pure tone audiometry conclusive of conductive hearing loss, with an air-bone gap ≥ 20 dB, 1 absent stapedial reflex on impedance audiometry, and normal appearance of the temporal bone on high-resolution computed tomography scans. And the exclusion criteria were history or presence of middle and external ear infection and pure tone audiometry conclusive of conductive hearing loss, with an air-bone gap < 20 dB.

### Points of Comparison

- Intraoperative timing (from infiltration of the local anesthetic agent in the external auditory canal until the placement of last piece of abgel).
- Surgical approach – in patients with wider external auditory canals, the transcanal approach was used; and in cases of a narrow canal, the postaural approach was preferred.
- Visualization of anatomical landmarks such as the long process of the incus and the anterior crura of the stapes.
- Extent of drilling of the posterosuperior part of the external auditory canal.
- Manipulation of the chorda tympani.
- Postoperative pain assessed through the Visual Analog Scale (VAS) 8 hours after surgery.
- Postoperative dizziness assessed 8 hours after surgery.
- Postoperative hearing assessment at the 4th week after surgery through pure tone audiometry, with a comparison with the preoperative hearing loss.

Both groups were compared using the Chi-squared test.

## Results

Table 1 shows the demographic characteristics of the sample. The mean age of the subjects in group A was 39.5 years, and, in group B, 38.5 years, which was not statistically significant ( *p* = 1).

**Table 1:** Demographic data of the study sample

Parameters		Group A (microscopic stapedotomy)	Group B (endoscopic stapedotomy)	*p* -value
Mean age (years)		39.5	38.9	1
Gender (n)	Male	4	3	0.63
	Female	6	7	

In both groups, there was a predominance of female subjects (6 in group A and 7 in group B), which was not statistically significant either ( *p* = 0.63).

Table 2 shows the comparison of intraoperative parameters. In group A, the mean intraoperative time was 54.5 minutes, while in group B it was 50.9 minutes, which was not statistically significant ( *p* = 1).

**Table 2:** Intraoperative parameters of the study sample

Parameters		Group A (microscopic stapedotomy)	Group B (endoscopic stapedotomy)	*p* -value
Mean intraoperative time (minutes)		54.5	50.9	1
Surgical approach (n)	Transcanal	6	10	0.025
	Postaural	4	0	
Anatomical landmarks visualized (n)	Anterior crura of the stapes	2	10	0.001
	Long process of the incus	7	10	0.06
Chorda tympani manipulated (n)		10	2	0.001
Need for drilling (n)		10	2	0.001

Regarding the approach, 6 patients in group A were submitted to the transcanal approach and 4, to the postaural approach. In group B, all patients were operated on via the transcanal approach. This was statistically significant ( *p* = 0.025).

The anterior crura of the stapes was visualized in all patients in group B, but only in 2 patients in group A, which was statistically significant ( *p* = 0.001).

The long process of the incus was visualized in 7 patients in group A, and in all subjects in group B, which was not statistically significant ( *p* = 0.06).

Manipulation of the chorda tympani was performed in all subjects in group A, but only in 2 patients in group B, which was statistically significant ( *p* = 0.001).

Drilling of the posterosuperior part of the external auditory canal was performed in all subjects in group A, and only in 2 subjects in group B, which was also statistically significant ( *p* = 0.001).

Table 3 shows the comparison of postoperative parameters. The mean postoperative pain score on the VAS was 5.8 in group A and 5.1 in group B, which was not statistically significant ( *p* = 1)

**Table 3:** Postoperative parameters of the study sample

Parameters		Group A (microscopic stapedotomy)	Group B (endoscopic stapedotomy)	*p* -value
Mean postoperative pain score on the Visual Analog Scale		5.8	5.1	1
Postoperative dizziness (n)		10	4	0.025
Postoperative hearing loss (air-bone gap) at the 4th week (n)	< 20 dB	5	6	0.23
	> 20 dB	5	4	

Postoperative dizziness assessed after 8 hours of surgery was present in all subjects in group A and only in 4 patients in group B, which was statistically significant ( *p* = 0.025)

The postoperative air-bone gap in the 4th week after surgery was lower than 20 dB in 5 patients in group A and in 6 patients in group B, and it was higher than 20 dB in 5 patients in group A and in 4 patients in group B, which was not statistically significant ( *p* = 0.23).

## Discussion

Stapedotomy, a surgical procedure to treat otosclerosis in which the traditional approach is microscopic, has undergone advancements with the introduction of endoscopic techniques.

In the current study, there was a predominance of female patients, which is in line with a study by Harikumar and Kumar, 3 in which there were 38 female and 22 male subjects.

In the present study, the mean intraoperative time for microscopic stapedotomy was 54.5 minutes, which was longer than the intraoperative time for endoscopic stapedotomy (50.9 minutes), a finding similar to that of the study by Migirov and Wolf. 6

In the current study as well as in the study by Kojima et al., 7 the transcanal approach was used only in microscopic stapedotomy. We also observed that visualization of the middle ear structures was better in the endoscopic procedure, which is in line with the findings by Nikolaos et al., 8 who stated that endoscopic stapedotomy provides more detailed visualization of the middle ear structures.

In the present study, there were higher levels of manipulation of the chorda tympani in microscopic stapedotomy, an observation similar to the one made by Mahendran et al., 9 in which manipulation of the chorda tympani was only performed in microscopic procedures.

We also observed that the need for drilling of the posterorsuperior part of the external auditory canal was minimal in endoscopic stapedotomy in the current study, which is in line with the study by Migirov and Wolf. 6

Regarding the levels of postoperative pain, they were higher in the microscopic group in the current study and in the study by Iannella et al. 10 As for the levels of postoperative dizziness, they were lower in the endoscopic group in the present study, and minimal in the same group in the study by Ataide et al. 11

According to present study, there was no significant difference in the postoperative air-bone gap between the the groups, a finding similar to the one made by Harikumar and Kumar. 3

Techniques such as canal straightening stitch or 3-mm otoendoscopy can also be used, but these techniques are surgeon- and institution-specific.

## Conclusion

**Fig. 1:**
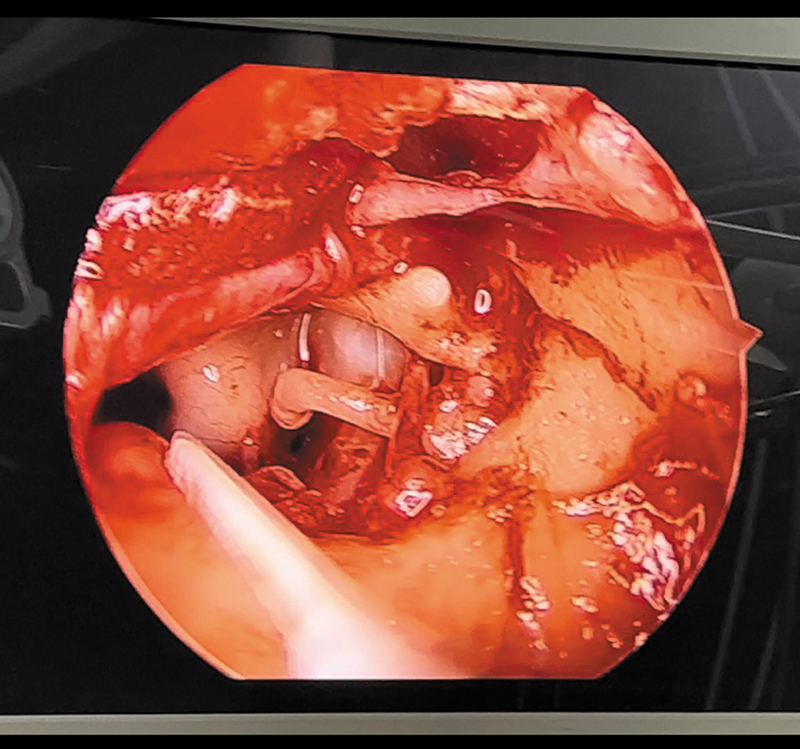
Middle-ear structures viewed from a 0° endoscope.

In conclusion, the findings of the present study shed light on the efficacy and safety of microscopic stapedotomy versus endoscopic stapedotomy in the treatment of otosclerosis. Through a meticulous examination and comparison of outcomes, it becomes evident that both techniques offer distinct advantages, but considerations must be made. Microscopic stapedotomy, with its established history and familiarity among surgeons, showcases consistent success rates. On the other hand, endoscopic stapedotomy emerges as a promising alternative, resulting in enhanced visualization of middle-ear structures, minimal need of drilling, lower levels of postoperative pain and dizziness, as well as a lower requirement of manipulation of the chorda tympani ( Fig. 1 ).

However, it is crucial to acknowledge the nuanced complexities and learning curves associated with each approach. Surgeon experience, patient-specific factors, and institutional resources all play pivotal roles in determining the optimal choice of technique. Furthermore, ongoing advancements in endoscopic instrumentation and surgical techniques may further shape the landscape of stapes surgery in the future.

Ultimately, the decision between microscopic and endoscopic stapedotomy should be made judiciously, considering the unique circumstances of each patient and the proficiency of the surgical team.
